# Supplementary material for: Association of the TTN, PDK4, and RNF207 mutations with dilated cardiomyopathy in Dobermanns from the United Kingdom
Source: PLoS One. 2025 Mar 13;20(3):e0319932. doi: 10.1371/journal.pone.0319932 (PMC11906055; doi:10.1371/journal.pone.0319932)
Supplement: Attachment — Submitted filename: reviewer responses submitted .docx [file pone.0319932.s002.docx]

Reviewer #1: Abstract

The abstract presents a study investigating the association of specific genetic mutations with dilated cardiomyopathy (DCM) in Dobermanns, focusing on three genes: TTN, PDK4, and RNF207. The authors hypothesized that the TTN and PDK4 mutations would not be associated with DCM in a UK cohort, while expecting an association for the RNF207 mutation. The study involved 74 dogs, including both controls and DCM-affected animals.

Undoubtedly, the hypothesis is clearly stated, providing a solid foundation for the study and the methods are appropriate for the research question. In addition, the results are presented in a concise manner, highlighting the key findings regarding allele frequencies and statistical significance and finally in conclusion you emphasize the relevance of the findings for screening and breeding practices, which is important for practical applications in veterinary genetics.

Thank you for your comments on our study, we are pleased to hear that you found the manuscript clearly written and of importance to veterinary genetics. We also thank you for your criticism on certain aspects and have addressed these in the document below. We believe the manuscript is now stronger because of your review and comments and would of course be happy to address any of the points further if additional clarity is required.

However, there are some areas for improvement that should be noticed. First of all, while the abstract mentions previous findings from US and European cohorts, it could benefit from a brief explanation of why these specific mutations were chosen and their relevance to DCM.

Thank you for the comment regarding the explanation of the specific mutations and why they were chosen, we have included a sentence to include this in brief detail (lines 19-21), however further elaboration is limited by the word count. The explanations are otherwise included in the introduction and discussion (introduction paragraphs 2 and 3, discussion paragraphs 2, 3 and 4).

Secondly, the sample size of 74 dogs may be considered small for genetic studies. A statement addressing the potential limitations of this sample size and its impact on the generalizability of the results would strengthen the abstract

Many thanks for this suggestion and we agree with this limitation, we have added to the abstract to highlight this as suggested (line 37).

Beyond that, while p-values are provided, additional context regarding the methodology for calculating odds ratios (OR) and confidence intervals (CI) would enhance transparency and reproducibility.

We agree this would add additional reproducibility however whilst we have included OR and CI in the abstract, the methodology for these are presented in the Materials and Methods rather than the abstract as the abstract is already over word-count with the other additional suggestions.

Eventually, it might be helpful to specify whether the mutations are novel or previously identified, as this could inform readers about the novelty of the findings

We have clarified that the *TTN* and *PDK4* variants were previously identified (line 35).

Overall, the abstract effectively summarizes the research conducted and presents meaningful findings regarding the association of RNF207 with DCM in Dobermanns in the UK. Addressing the areas for improvement could enhance clarity and provide a more comprehensive understanding of the study's significance and limitations. 

Introduction
The introduction provides a thorough overview of dilated cardiomyopathy (DCM) in dogs, particularly focusing on Dobermanns. It outlines the disease's prevalence, genetic implications, and previous research findings related to specific gene mutations. The authors set the stage for their hypothesis regarding the association of TTN, PDK4, and RNF207 with DCM in a UK cohort.

The introduction effectively establishes the significance of DCM in Dobermanns, including its prevalence and impact on health, which helps readers understand the importance of the study.

The authors provide a well-rounded review of existing literature, discussing both US and European studies that have explored genetic associations with DCM. This contextualizes their research within the broader scientific conversation.

However, here are some specific comments and suggestions to enhance the introduction further:
Several studies, including the RNF207 gene's potential role in DCM, are mentioned but lack detailed explanations

We have added further explanation over the potential role of RNF207 in DCM to improve clarity and context for the reader as suggested. The lines 63-68 now read ‘*RNF207* encodes a heart-specific really interesting new gene (RING) finger protein that has been shown to interact with the voltage-dependent anion channel (VDAC) 1, regulate the repolarizing channel HERG and is involved with pathological cardiac hypertrophy [14–16]. Therefore, variants in *RNF207* may result in QT prolongation, shortened action potential duration and altered energy metabolism, which may play a role in DCM.’

The transition between previous findings and the hypothesis could be smoother. It is suggested to include specific research objectives or questions to guide the study beyond gene sequencing for a clearer narrative flow.

Thank you for the suggestion, we have added the following lines to the paragraph, which hopefully makes the transition smoother: ‘The previously reported *TTN* and *PDK4* have failed to show an association with DCM in European Dobermanns and the newly discovered *RNF207* mutation has not been studied in a UK cohort of Dobermanns.’ (lines 70-72).

Materials and Methods

Here are some comments and suggestions regarding the "Materials and Methods" section of your study:

The multicenter, retrospective case-control design is clearly stated, which helps readers understand the framework of the study.

Mentioning the ethical review committee and approval number adds credibility and shows adherence to ethical standards.

The detailed diagnostic criteria for DCM, including specific measurements and thresholds, is well-articulated and demonstrates rigor in identifying cases.

The DNA extraction process is adequately described, including the use of specific kits and methods for quality assessment.

There is no mention of statistical methods used for analysis. This is crucial for understanding how results will be interpreted.

Thank you for the positive review of M&Ms section and we are pleased to hear that our methods were clear. There is a section on statistical methods used (lines 154 – 162), please let us know if this requires further clarity.

Results

With regard to positive aspects: the study provides a detailed description of the study population, including the selection criteria and demographic information (e.g., age, sex distribution). This enhances transparency and reproducibility.
The results present specific echocardiographic measurements that highlight significant differences between DCM and control groups. This is crucial for understanding the physiological implications of DCM in Dobermanns.

The inclusion of allele frequencies for the TTN, PDK4, and RNF207 variants adds valuable genetic context to the study, which is important for understanding potential genetic predispositions to DCM.

When it comes to negative points: the study's sample size (74 Dobermanns) may limit the generalizability of the findings. Small sample sizes can lead to type II errors, where true associations are missed.

We agree with this point, and unfortunately due to the retrospective nature of the study there were many samples in the DNA archive that we could not rely upon to be robust for including in the study, therefore we opted to include only cases over a certain age with a confirmed diagnosis to obtain the cleanest case and control population. We have added these limitations to our discussion so it now reads: ‘The sample size was small for a genetics study, which may limit the generalizability of the results, and lead to type II errors where true associations are missed, which are important factors when interpreting our findings. Studies that are prospective using a larger population of Dobermanns would therefore be needed to confirm these findings.’ (lines 361-365).

Lack of Longitudinal Data: The cross-sectional nature of the study does not allow for insights into disease progression or the effects of potential interventions over time.

We agree that longitudinal data would be powerful to assess disease progression and effects of any interventions, however the aim of the study was to initially assess if there was credibility in the genetic variants that have been previously reported. In our limitations we mention the age cut-off of 7 years may mean some control dogs would go on to develop DCM. A very useful study to perform would be to genotype young Dobermanns and follow them over time, however given the limitation of veterinary medicine with regards to cost, owner compliance etc. this study has not been performed, but with more studies such as ours indicating which mutations need to be assessed further, hopefully future studies will provide this data.

Limited Genetic Analysis: While allele frequencies are reported, the association analyses do not seem to demonstrate a significant link between genetic variants and DCM, which may warrant further investigation or different analytical approaches.

We agree that our limited genetic analyses do not highlight strong links between variants and DCM, however there have been previously very well powered studies that used advanced genetic association techniques to find the variants we have analysed in our study, therefore it is unlikely that with our smaller sample size, additional analyses would have identified any new genetic variants. (Niskanen JE, Ohlsson Å, Ljungvall I, Drögemüller M, Ernst RF, Dooijes D, et al. Identification of novel genetic risk factors of dilated cardiomyopathy: from canine to human. Genome Med. 2023;15: 73. doi:10.1186/s13073-023-01221-3).

Practical Comments:

Consider Increasing Sample Size: Future studies could benefit from a larger sample size to enhance the power of statistical analyses and improve the reliability of findings regarding genetic associations with DCM.

Thank you for the comment and we agree that a greater sample size would be beneficial. However, in veterinary studies our sample size is similar or greater than those in the previously reported US studies. For example, Meurs et al. 2012 that identified the PDK4 mutation published in Human Genetics used 66 Dobermanns, and the 2020 paper looking at TTN and PDK4 together analysed 48 Dobermanns. We feel that despite the limitations mentioned, our study shows that the RNF207 variant appears associated with DCM in a UK population of Dobermanns which was the main objective of the study. We hope this result will support future prospective studies involving a greater sample size.

Longitudinal Studies: Conducting longitudinal studies could provide insights into how DCM progresses over time in Dobermanns and how genetic factors might influence this progression.

We agree that longitudinal studies following those genetically at risk would be beneficial, and one we are considering. However due to the numbers required this would need a multicentre approach to recruit the number of cases and significant funding (due to the age-related penetrance of the disease and the need to wait 10-15 years until all the dogs had died). Our aim was first to analyse retrospective data we had available to first ascertain which mutations are likely to be associated and would benefit from longitudinal studies.

Broaden Genetic Analysis: Expanding the analysis to include additional genetic variants or employing whole-genome sequencing could uncover more significant associations with DCM.

We agree this would be a powerful approach, but this was not the aim of the study, especially considering the small sample size. Our study was specifically looking at variants that had already been identified using the techniques you describe.

Discussion
The study commences with a well-defined objective, which effectively sets the stage for the ensuing findings and provides a solid context for the results. The authors make pertinent comparisons between human and canine dilated cardiomyopathy (DCM), underscoring the differences in genetic links and their implications for understanding the disease across both species. This enriches the discussion and highlights the importance of canine models in human research.

The authors successfully place their findings within the wider framework of genetic research on DCM, referencing prior studies and established genetic mutations related to the disease in both dogs and humans.

However, there are some points that should be addressed. Although the association with RNF207 is acknowledged, there is insufficient exploration of its biological significance or the possible mechanisms through which it may impact DCM in Dobermanns. A more in-depth discussion regarding how this variant could influence cardiac function would enhance this section.

Thank you for this comment, although the exact role of RNF207 in cardiac function and DCM is still being explored, we have added additional detail to this section so it now reads (lines 320 – 343): ‘*RNF207* encodes the really interesting new gene (RING)-finger protein 207 (RNF207), which has proposed actions in regulating the cardiac action potential and is involved in pathways known to regulate cardiac hypertrophy [15,16]. Additionally, it has been shown in humans that abnormal function of the RNF207 protein is implicated in or can worsen the expression of long-QT syndrome, known to result in ventricular arrhythmias causing syncope and sudden death [36,37]. Also it has been shown that RNF207 plays an important role in the production of ATP in cardiomyocytes, and that dysfunction in this protein may worsen the development of congestive heart failure [14]. In a study using canine myocardium, researchers discovered that RNF207 is expressed in the intercalated discs between cardiomyocytes (as well as in the cytoplasm and perinuclearly) [11] . Since the gap junctions present in the intercalated discs are vital for ion passage between cells and coordinated cardiac contraction, disruption to this region may lead to impaired cardiac function. Other studies have shown that alteration to the intercalated discs is directly associated with the development of DCM [38–40] . Additionally, immunohistochemistry in a dog that was homozygous for the *RNF207* variant showed marked cellular mosaicism for RNF207, indicating the variant alters expression of the protein that may affect cardiac function by the previously mentioned mechanisms [11]. Additionally, due to the role of *RNF207* in ATP production in cardiomyocytes, it is also possible that mutations in *RNF207* could influence the expression of DCM in Dobermanns as opposed to being directly causal, and this would also explain why not all Dobermanns with DCM carry this variant.’

In addition, the discussion fails to provide explicit recommendations for future research or how the findings could guide breeding practices, genetic screening, or clinical interventions. This omission may leave readers seeking more definitive next steps

We have updated our recommendation on further research to me more specific so this reads ‘Further research is warranted that validates the findings for the variant in *RNF207* using larger sample sizes with prospective study design and following dogs longitudinally to assess outcome in patients with certain genotypes. Additional research could then assess whether early detection of at-risk individuals and therefore earlier clinical intervention (such as more regular screening or treatment with disease-modifying drugs) may improve outcomes in this patient population.’ (lines 383 - 389).

Reviewer #2: This well-written article makes a valuable contribution to animal cardiogenetics, offering insights relevant to both dogs and humans with dilated cardiomyopathy. Below are a few points that may enhance the manuscript's clarity and impact.

Thank you for your comments on our study, we are pleased to hear that you found the manuscript well-written and insightful. We also thank you for your criticism on certain aspects and have addressed these in the document below. We believe the manuscript is now stronger because of your review and comments and would of course be happy to address any of the points further if additional clarity is required.

1. Have the authors considered whether regional breeding practices might influence the prevalence of certain DCM-associated mutations within the UK cohort compared to other European or U.S. populations? This could be expanded by discussing the possibility of founder effects, especially given the historically limited genetic diversity within the Dobermann breed.

Thank you very much for this comment, and a particularly interesting topic given the geographically distinct populations of Dobermanns with limited genetic pools, which we included in our discussion. Previous studies on Dobermann populations identified four major cohorts but these were classified into large geographical locations. The possibility that regional breeding practices in the UK could have caused the differences is certainly possible, and we have expanded our discussion to raise note of this possibility. Unfortunately, our sample size of Dobermanns from all over the UK was not large enough to reliably detect founder effects. However, the ease of buccal swabs and the prospect of an expanded future study we could certainly assess the effect of geographical location, and this would be interesting. We have added the clarifications to the discussion such that it reads: ‘It is possible that regional breeding practices in the UK may have influenced the prevalence of DCM-associated mutations compared to other populations and given the limited genetic diversity of Dobermanns in the UK there is the possibility of founder effects. However, our study did not have a sufficient sample size to assess if geographical location affected a genotype-phenotype relationship. (lines 284 – 288) and ‘Additionally, assessment of geographical location within the UK could help decipher whether there are founder effects resulting in differences between geographically distinct Dobermann populations.’ (lines 368 – 371).

2. Could the authors clarify if there were any specific subgroups or characteristics within the DCM cohort that influenced the strength of the RNF207 association? Were there phenotype variations based on the homozygous presence of the RNF207 variant?

Thank you for the comment and useful suggestion. Given the possibility that the variant in RNF207 may play a disease modifying rather than disease causing role, we agree this would be valuable information. Unfortunately, due to the retrospective nature of the study (meaning some missing data), and the low numbers of cases that were homozygous for the mutation, it wasn’t possible to draw any firm additional conclusions from the data we obtained. We did look at the effect on age at diagnosis, however this was not significant. We have added lines 340 - 343 to highlight this. Ideally, we would need a prospective study that was appropriately powered to be able to identify these associations or effect on phenotype, for example if the phenotype more severe or more arrhythmogenic.

3. Did the authors perform a power analysis to determine if this sample size was adequate to detect statistically significant differences, especially given the allele frequencies presented? It would strengthen the study to discuss the potential limitations related to sample size, especially for low-frequency variants.

Thank you for this comment, we did not perform a power analysis for this study, however based our sample size on previous studies in the US which showed an association between the variants and the development in DCM with similar or smaller sample sizes. Given the allele frequencies in our population were very similar to those previous studies, we had assumed that if the same level of association existed, our study would have verified those findings. However, we do agree the sample size is small for a genetic study (and unfortunately a limitation in general with veterinary studies), and have highlighted that this may lead to type II errors in the limitations section of the discussion (lines 361 - 363), so this now reads: ‘The sample size was small for a genetics study, which may limit the generalizability of the results, and lead to type II errors where true associations are missed, which are important factors when interpreting our findings.

4. Are chi-square tests appropriate given the sample size, particularly for rare genotype combinations? Did the authors consider alternative statistical methods (e.g., Fisher’s exact test) for smaller samples?

Thank you for the comment. Since we compared allele frequencies between cases and controls, no cell had a frequency of <5 and therefore we chose to use Chi-Square test. We re-ran the statistics using Fisher’s exact test and there remained the statistical difference for *RNF207* but no difference for either of the other 2 variants.

5. While the abstract suggests some genetic differences across populations, it would be valuable if limitations regarding population sampling, genetic heterogeneity, and broader applicability were noted. Could the authors acknowledge any potential biases in their cohort selection or limitations regarding sample representativeness?

Many thanks for the suggestion and we agree with this limitation, we have added to the limitation section so this now reads ‘Additionally, the small sample size and possibility that sampling did not represent the UK population as a whole, there is potential for bias in the sampling method (which relied on owners presenting dogs for examination rather than random sampling) and representativeness of the sample to the UK as a whole.’ (lines 365 – 368).

6. Could the authors draw further parallels between the mechanisms of action of these mutations in humans and dogs, especially concerning how TTN and RNF207 may differentially influence DCM phenotypes?

Thank you for this comment. We have expanded the section on the mechanisms of action for RNF207 so this section now reads: ‘In a study using canine myocardium, researchers discovered that RNF207 is expressed in the intercalated discs between cardiomyocytes (as well as in the cytoplasm and perinuclearly) [11] . Since the gap junctions present in the intercalated discs are vital for ion passage between cells and coordinated cardiac contraction, disruption to this region may lead to impaired cardiac function. Other studies have shown that alteration to the intercalated discs is directly associated with the development of DCM [38–40] . Additionally, immunohistochemistry in a dog that was homozygous for the *RNF207* variant showed marked cellular mosaicism for RNF207, indicating the variant alters expression of the protein that may affect cardiac function by the previously mentioned mechanisms [11]. Additionally, due to the role of *RNF207* in ATP production in cardiomyocytes, it is also possible that mutations in *RNF207* could influence the expression of DCM in Dobermanns as opposed to being directly causal, and this would also explain why not all Dobermanns with DCM carry this variant. It is therefore also possible that there are specific subgroups or characteristics of the dogs with the *RNF207* variant that may influence the strength of the association with DCM, or specific phenotypic features, that were unable to be identified in this study and would benefit from future investigation.’ (lines 328 – 343)

We have also expanded the section on the titin mutation so this now reads: ‘Specifically, titin acts as a so-called molecular spring, where it contributes to the stiffness of the myocardium as well as contraction [35] . In humans, variants in *TTN* that result in a truncated protein account for 20-25% of DCM cases where a genetic mutation is found [33,36]. They are reported to occur throughout the titin gene, with most being found in the region of titin that spans the A-band of the sarcomere [37].  The variant in *TTN* that was discovered by Meurs *et al.* was instead located in an immunoglobulin-like domain of titin called I71 and is part of one of the spring elements that make up the protein [38]. It is hypothesised that the missense mutation in this I71 domain may unfold the region or make it easier to unfold during diastole, which may make it sensitive to degradation and affect passive and/or active tension [7].’ (lines 296 – 305).
